# Faltwerk: a library for spatial exploratory data analysis of protein structures

**DOI:** 10.1093/bioadv/vbad007

**Published:** 2023-01-23

**Authors:** Adrian Viehweger

**Affiliations:** Institute of Medical Microbiology and Virology, University of Leipzig Medical Center, Leipzig 04103, Germany; Institute of Human Genetics, University of Leipzig Medical Center, Leipzig 04103, Germany

## Abstract

**Summary:**

Proteins are fundamental building blocks of life and are investigated in a broad range of scientific fields, especially in the context of recent progress using *in silico* structure prediction models and the surge of resulting protein structures in public databases. However, exploratory data analysis of these proteins can be slow because of the need for several methods, ranging from geometric and spatial analysis to visualization. The Python library faltwerk provides an integrated toolkit to perform explorative work with rapid feedback. This toolkit includes support for protein complexes, spatial analysis (point density or spatial autocorrelation), ligand binding site prediction and an intuitive visualization interface based on the grammar of graphics.

**Availability and implementation:**

faltwerk is distributed under the permissive BSD-3 open source license. Source code and documentation, including an extensive common-use case tutorial, can be found at github.com/phiweger/faltwerk; binaries are available from the pypi repository.

## 1 Introduction

Exploratory data analysis is often used to generate new hypotheses. Such exploratory work is more effective when it takes little time and effort to create new viewpoints on the data. However, subanalyses often add substantial friction and prolong feedback, especially with multimodal data such as protein structures. Here, many tracks of evidence need to be integrated: the binding of ligands and other proteins occurs at specific residues and is determined by physicochemical features such as solvent access and electrostatic forces, which in turn can be affected by mutations (Bhattacharya *et al.*, 2017), data which usually has been collected from several individuals of a population of interest (species, patients). More complexity is added by the fact that proteins fold into three-dimensional structures, which in their evolutionary history are more conserved than the underlying linear amino acid sequence (Illergård *et al.*, 2009). In fact, this folding allows a pair of residues to be far apart on the protein sequence but very close in three dimensions. Because structure determines function, it can be helpful to add, for example, spatial features. Mutations have been observed to sometimes cluster and thus mark functionally important parts of a protein, both in disease (Kamburov *et al.*, 2015; Li *et al.*, 2022; Sivley *et al.*, 2018) as during environmental adaptation (Barber and Elde, 2014; Kiefl *et al.*, 2023; Slodkowicz and Goldman, 2020). It is these spatial patterns that Tobler’s first law of geography addresses: ‘Everything is related to everything else, but near things are more related than distant things’ (Tobler, 1970). For example, Barber and Elde (2014) observed the asymmetric clustering of substitutions in a specific region of the protein transferrin, which led to the hypothesis and subsequent experimental validation that the observed mutation pattern likely derives from coevolution. We will use this example as a use case below.

Three main approaches have been pursued to analyze spatial signals on proteins. First, mapped entities (protein residues) can be grouped based on pairwise distance, sometimes referred to as point-density analysis (Meyer *et al.*, 2016; Ryslik *et al.*, 2014). This approach relies solely on atomic coordinates. Popular algorithms include Markov chain clustering (MCL) (Enright *et al.*, 2002) and HDBSCAN (McInnes and Healy, 2017). Second, one can aggregate non-spatial information attached to these coordinates using a spherical window sliding along the (folded) protein sequence from N- to C-terminus (Hicks *et al.*, 2019; Silk *et al.*, 2021). Third, one can test how non-spacial features are distributed and whether they form ‘hotspots’ (Fujimoto *et al.*, 2016; Kamburov *et al.*, 2015; Slodkowicz and Goldman, 2020; Tokheim *et al.*, 2016; Turner *et al.*, 2015). Because most of these methods compare the residues in local ‘patches’ of protein against randomly permuted ones, multiple comparison correction is required (Benjamini and Hochberg, 1995). Many of the spatial methods used for protein analysis are *ad hoc* variations on more ‘classical’ ones from the geographical sciences, namely *Ripley’s K* (Sivley *et al.*, 2018), *Moran’s I*, and the *Getis-Ord G* family of statistics. Robust implementations exist, in contrast to most studies, for which either no code was available or which would require significant refactoring to use outside of the original work.

To our knowledge, faltwerk is the first library that allows analyses across all three spatial analysis types. While one can use other tools like anvio ‘structure’ (Eren *et al.*, 2021; Kiefl *et al.*, 2023) to explore protein structures, none includes methods for spatial data analysis at the time of writing. However, in light of the steep increase of available protein structures, mainly driven by *AlphaFold v2* (Jumper *et al.*, 2021) but likely a more general trend (Jones and Thornton, 2022; Mirdita *et al.*, 2022), faltwerk will be a valuable tool for many users in computational and molecular biology.

## 2 Functionality

faltwerk is a framework to facilitate exploratory data analysis of proteins. It offers many functions for handling protein structures and complexes, including easy loading, subsetting protein complexes, and annotating protein domains and conserved sites. By centering on exploration, a central part is a well-designed API to visualize the structures, inspired by the grammar of graphics (Wilkinson, 2005), a concept that allows the layering of graphical objects. Protein structures and complexes render in jupyter and colab notebooks, ideal for exploratory work or for sharing such analyses. Unlike stand-alone applications in the protein space, faltwerk is a python library that integrates well into existing tools and workflows for high throughput processing and is distributed under a permissive BSD-3 license. Besides these features, which are required from a broadly applicable library, faltwerk also includes sophisticated functions to explore protein structures spatially. While this might seem like a niche at the moment, we hypothesize that with the enormous growth in protein structure predictions, such analyses will become more mainstream. For example, faltwerk allows spatial clustering of sites under positive evolutionary selection. When integrated into a workflow, thousands of proteins can be screened to identify potential targets of immune processes or coevolution (Shultz and Sackton, 2019), not just on the linear sequence, but in three dimensions (Gao *et al.*, 2017; Kamburov *et al.*, 2015).

**Fig. 1.**
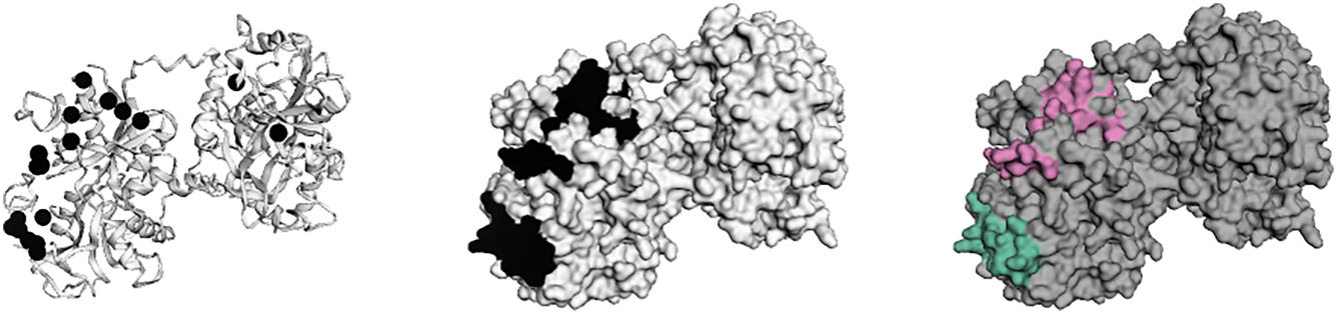
Visualization of residues under positive selection identified by Barber and Elde (2014) in human transferrin (leftmost panel). Note the asymmetric distribution on the C-terminal lobe of the otherwise symmetric protein. This pattern led them to a hypothesis that co-evolution with an iron-scavenging receptor of the bacterium *Neisseria meningititis* was responsible, which was shown to be correct using *in vitro* experiments. A hotspot analysis (middle panel) marks this region and allows co-localization with the bacterial receptor binding interface (not shown); note how two positions on the N-terminal lobe do not result in hotspots. In the rightmost panel, the hotspot residues are segmented (clustered) using HDBSCAN into two clusters. In an automated workflow, one could now run a regression model for each of those on the assumption that distinct protein features drive the respective pattern

Specifically, the library implements standard parsers for files in the common PDB format and can also be used to parse and explore protein complexes. The library can handle files in PDB format stored as strings and provides access to commonly used biopython structure objects, facilitating integration with existing code bases. However, faltwerk adds objects on top of these standard ones to facilitate work with protein complexes and *in silico* predictions. For example, to remove chains A and B from a protein complex, one can use the following concise syntax: cx = Complex(path); cx =- “AB”. A custom AlphaFold object handles additional metadata generated during prediction such as *pLDDT*, a metric that estimates prediction quality (Jumper *et al.*, 2021). Structures can be aligned directly from within faltwerk, which wraps foldseek (van Kempen *et al.*, 2022) for this purpose. Furthermore, several functions allow to explore the geometry of the protein structure and extract annotations, such as per-residue distance to the binding site in a protein complex. Such relations are relevant because enrichment of mutations at protein-protein interaction interfaces has been reported (Slodkowicz and Goldman, 2020). The result can then be visualized using an intuitive API inspired by the grammar of graphics (Wilkinson, 2005) using the 3Dmol.js library for rendering (Rego and Koes, 2015). In short, a layout is specified, onto which graphics can be layered. Optionally, only a subset of the data can be selected, e.g. when visualizing mutations or residues that are part of an active site. This approach allows features to be explored quickly and in relation to one another (Fig. 1). Note that faltwerk acts as a layer of abstraction or ‘glue’ between many well-tested functions for protein exploration. For benchmarks, we refer the reader to these respective libraries and their associated method descriptions, e.g. pysal (github.com/pysal) or hdbscan (McInnes and Healy, 2017).

For spatial exploration, we implement (i) point density clustering using HDBSCAN (McInnes and Healy, 2017) (default) and MCL (Enright *et al.*, 2002). While these algorithms require very different implementations, we expose a common interface, and users can seamlessly add, switch and compare methods. Furthermore, we implement (ii) a sliding spherical window in which protein features can be aggregated. Last, hotspots can be identified using (iii) local spatial autocorrelation using either the *Getis-Ord G* family of metrics (Getis and Ord, 1992) (default) or *Moran’s I* (Moran, 1950), including multiple hypothesis correction using a specified false discovery rate (Benjamini and Hochberg, 1995). Again, a common interface makes switching between methods trivial. In contrast to previous work, faltwerk allows exploration of spatial methods and individual components such as different distance functions. For example, a fixed radius around a residue of interest is often used to define neighbors. In our library, users can experiment with other functions, such as weights that decay with distance, where the neighborhood is defined on a continuous scale. Lastly, faltwerk implements protein domain and ligand binding site prediction, using the approach from Kobren and Singh (2019). Here, we rely on custom code written by E. Kiefl (https://merenlab.org/2020/07/22/interacdome/). For subsequent analyses, the library provides methods to export protein annotations as a data frame which can then be used without modification with subsequent tools. Users might follow up using spatial regression or machine and deep learning to identify features that might be predictive for sites of interest, such as those under positive selection. Below, we provide an example and the subsequent visualization of a hotspot analysis and clustering of residues under positive selection identified by Barber and Elde (2014):

*# Skipping imports*

*# Load model and residues under positive selection from Barber et al., Science, 2014*

model = Fold(”/path/ to/ structure.pdb”)

original = [152, 252, 381, . . .]

positive = [1 **if i in** original else 0 **for i in range** ( **len** (model))]

*# (1) Spatial autocorrelation , defaults to Getis–Ord metric*

hotspots = find_hotspots(model , positive , false_discovery_rate = 0.05)

*# (2) Point density analysis , defaults to HDBSCAN*

clusters = cluster(model , hotspots , min_cluster_size = 5)

model.annotate_many_({”positive”: positive, ”hotspots”: hotspots , ”clusters”: clusters})

ly = Layout(model , panel_size=(200, 200), grid = (1, 3), linked=True)

mask = ly.select(residues=positive, elements = [”CA”], chain=”A”)

ly.geom_ribbon(color = ”#ffffff”)

ly.geom_sphere(selection = mask, color=”black”)

ly.geom_surface(”hotspots”, palette=”binary”, panel=(0, 1))

ly.geom_surface(”clusters”, palette=”Set2_r”, panel=(0, 2))

ly.render().show()

## Data Availability

No new data were generated or analyzed in support of this research.
